# Insinuating

**Published:** 1858-01

**Authors:** 


### INSINUATING.

The *Sacramento Age* says: "A writer in one of our standard medicinal journals inquires why women are more liable to take cold than men? Indeed we do not know, and wouldn't be ungallant; but Dr. Hall says, that one of the best ways to avoid taking cold is, '*To keep the mouth shut.*'"

